# Nurses v. Trade Unions

**Published:** 1919-06-14

**Authors:** 


### THE EDITOR'S LETTER-BOX.

### Nurses v. Trade Unions.

*To the Editor of* THE HOSPITAL.

SIR,—Lord Ampthill said in the House of Lords on May 27, when the College Bill for State Registration was under discussion, that if nurses were not granted registration they would be forced to form a trades union. It is hoped and believed that no real nurse would ever agree to that. Firstly, because nursing is not a trade; secondly, the work is of such a nature, involving as it does the health and the lives of others, that it must always be considered before the worker. The work always has and always must come first; the interests and the welfare of the nurse second. That is the unwritten law of nurses, made and maintained by all that are worthy of the name. Trades unions, involving as they do strikes and limitations in which all must participate, would entirely destroy that high ideal of self-sacrifice and devotion which has made nursing what it is, the best and noblest profession open to women. It still is, but it never would be again once a trades union was formed. Nurses might possibly receive some *material* benefit, but the profession of nursing would receive an injury from which it would never recover. If nurses can get State registration, let them be glad and rejoice; if they cannot, let them go on as before. Trades unions are for trades.

Also there is no need for Lord Ampthill to fear that nurses "will throw themselves into the arms of the Labour Party." They would indeed prefer to trust their interests to the House of Lords, who discussed the Bill from both

(Continued on page 278.)

### THE EDITOR'S LETTER-BOX—*(continued from p. 275).*

sides with such sympathy and friendliness and such unusually true understanding of the position of nurses. Lord Russell said that, judging from his post-bag and the Lobby on the occasion of the debate, the majority of nurses did not appear to be in favour of the College Bill. To say what is strictly true in answer to that it must be admitted that the majority of nurses, though mildly interested, do not much care either way. There are enthusiastic partisans on both sides; the more heated, perhaps, are those against the College. They care so much that they will wait for hours to get into the Houses of Parliament, attend meeting after meeting, write letter after letter; will, if they are not careful, run some danger of destroying within themselves "that curious electric spark of sympathy," which cannot be defined and which Viscount Sandhurst described so admirably as the best and most subtle quality of the real nurse. That quality grows and expands, is strengthened and perfected in an atmosphere of steady "carrying on" in the quiet enthusiasm and growing absorption in the work for the work's sake, varied by a rest-time occupied in the gentler forms of recreation. It is certainly weakened and finally destroyed by much standing about in lobbies, attending hot and crowded meetings, energetically supporting or opposing Acts of Parliament.

It was just the same before women had the vote. The minority fought for the cause, the majority cared not at all or were only mildly interested, and in the meantime got on with the work. This is, as was said before, possibly deplorable but unquestionably true.—Yours faithfully,

